# Metformin in Pregnancy: Mechanisms and Clinical Applications

**DOI:** 10.3390/ijms19071954

**Published:** 2018-07-04

**Authors:** Steve Hyer, Jyoti Balani, Hassan Shehata

**Affiliations:** 1 Department of Endocrinology, Epsom and St. Helier University Hospitals NHS Trust, Wrythe Lane, Carshalton SM5 1AA, Surrey, UK; steve.hyer@nhs.net; 2 Department of Maternal Medicine, Epsom and St. Helier University Hospitals NHS Trust, Wrythe Lane, Carshalton SM5 1AA, Surrey, UK; hassan.shehata@nhs.net

**Keywords:** metformin, pregnancy, gestational diabetes, polycystic ovarian syndrome, type 2 diabetes, obesity

## Abstract

Metformin use in pregnancy is increasing worldwide as randomised controlled trial (RCT) evidence is emerging demonstrating its safety and efficacy. The Metformin in Gestational Diabetes (MiG) RCT changed practice in many countries demonstrating that metformin had similar pregnancy outcomes to insulin therapy with less maternal weight gain and a high degree of patient acceptability. A multicentre RCT is currently assessing the addition of metformin to insulin in pregnant women with type 2 diabetes. RCT evidence is also available for the use of metformin in pregnancy for women with Polycystic Ovarian Syndrome and for nondiabetic women with obesity. No evidence of an increase in congenital malformations or miscarriages has been observed even when metformin is started before pregnancy and continued to term. Body composition and metabolic outcomes at two, seven, and nine years have now been reported for the offspring of mothers treated in the MiG study. In this review, we will briefly discuss the action of metformin and then consider the evidence from the key clinical trials.

## 1. Introduction

In recent years, metformin has gained acceptance as a safe, effective and rational option for reducing insulin resistance in pregnant women with type 2 diabetes, gestational diabetes (GDM) or polycystic ovarian syndrome (PCOS). It may also provide benefit in obese non-diabetic women during pregnancy. In the UK, the National Institute for Health and Care Excellence (NICE) recommends that women with gestational diabetes should be offered metformin if blood glucose targets are not met with diet and exercise within 1–2 weeks [1]. The Scottish Scottish Intercollegiate Guidelines Network (SIGN) guidelines recommend that metformin or glibenclamide may be considered as initial pharmacological glucose lowering treatment in GDM [2]. Diabetes Canada states that metformin may be used as an alternative to insulin adding that women should be informed that metformin crosses the placenta, longer term studies are not yet available and the addition of insulin is necessary in approximately 40% to achieve adequate glycaemic control [3]. By contrast, the American Diabetes Association (ADA) states that insulin is the first line of treatment for GDM [4]. However, it classifies metformin as Category B and refers to evidence of safety and efficacy from randomised trials whilst noting that long term safety data for offspring is lacking [4]. We will briefly consider the action of metformin and then review the major clinical trials of metformin use in pregnancy including its use in obese non-diabetic women and as an addition to insulin in type 2 diabetes. We conclude by looking at the potential impact of in utero exposure in the offspring of mothers receiving metformin in pregnancy.

## 2. Mechanisms of Action of Metformin

Metformin improves insulin sensitivity, reduces hepatic gluconeogenesis, and increases peripheral glucose uptake [5,6]. It reduces fasting serum insulin by 40% (hence the risk of hypoglycaemia is minimal) and leads to a mean weight reduction of 5.8% [7]. The improvement in insulin sensitivity is mediated via several mechanisms including increased insulin receptor tyrosine kinase activity, enhanced glycogen synthesis, reduces the rate of glycogenolysis, decreased activity of hepatic glucose-6-phosphatase, and an increase in the recruitment and activity of GLUT4 glucose transporters [5,8]. Metformin also stimulates glucagon-like-peptide-1 (GLP-1) release thereby enhancing insulin secretion [6]. It also has profound effects on adipose tissue [9]. Metformin promotes the re-esterification of free fatty acids and inhibits lipolysis. The suppression of fatty acid oxidation results in a reduction in hypertriglyceridemia, thus reducing the energy supply for gluconeogenesis. This is associated with decreased synthesis and increased clearance of Very Low Density Lipoprotein (VLDL). The inhibition of lipolysis and reduction in triglyceride levels may contribute to improved insulin sensitivity through reduced lipotoxicity [9].

At the molecular level, these effects are attributed to activation by metformin of hepatic AMP-activated protein kinase (AMPK), a major cell regulator of lipid and glucose metabolism [9]. AMPK activation leads to a reduction in acetyl-CoA carboxylase (ACC) activity which in turn reduces fatty acid oxidation and suppresses lipogenic enzymes. AMPK activation in skeletal muscle also provides a mechanism for increased peripheral glucose uptake. AMPK plays an important role in the regulation of GLUT4 glucose transporters. Increase glucose uptake into muscle is associated with increased glycogen synthetase activity and glycogen storage [9].

Several mechanisms have been proposed for the gastrointestinal effects of metformin including delayed intestinal glucose absorption, augmented lactate production by enterocytes, enhanced secretion of gastrointestinal hormones containing GLP-1, and effects on bile acid metabolism [10]. Metformin has been shown to favourably alter intestinal microbiota [10]. It has been suggested that the primary action of metformin is at the level of the gut [10].

In a long term prospective study of type 2 diabetes, metformin was associated with reduced cardiovascular and all-cause mortality compared to sulphonylureas and insulin despite similar glycaemic control (UKPDS) [11]. This effect may be mediated by activation of the RISK pathway via increased AMPK activity [9]. In addition, metformin inhibits formation of advanced glycation end products (AGEs) which might be important in reducing diabetic vascular complications [12]. Metformin reduces AGE formation directly via an insulin dependent mechanism and indirectly by a reduction in hyperglycaemia.

At the cellular level, metformin, a positively charged molecule, is transported across the mitochondrial membrane by organic cation transporters (OCTs). In the context of pregnancy, it is important to note that the placenta expresses many isoforms of OCTs and hence metformin readily crosses the placenta [13]. Transport across the placenta into the developing foetus raises concerns regarding potential adverse effects on foetal development. It is not known whether the human embryo expresses OCTs but pre-implantation human embryos have low mitochondrial content and hence may be unresponsive to metformin [14].

In experimental mouse models, maternal diabetes leading to congenital abnormalities is associated with elevated embryo AMPK activity [15]. However, investigators have not been able to demonstrate increased embryo activity or congenital malformations after metformin administration at doses that stimulated maternal liver AMPK [16]. Metformin does stimulate AMPK activity in cell cultures of mouse embryonic stem cells in vitro but the significance of this finding is unclear given the lack of effect on mouse embryos in vivo.

## 3. Clinical Applications

### Metformin in Obese Non-Diabetic Pregnant Women 

Maternal obesity is associated with adverse pregnancy outcomes including an increased risk of gestational diabetes, preeclampsia, and macrosomia [17,18,19,20,21]. Sebire et al. [21], in a study of 287,213 pregnancies in London, reported odds ratio (OR) for obese women compared to those with normal Body Mass Index (BMI): GDM (OR: 3.6), PET (OR: 2.1), LGA (OR: 2.36). Whilst the mechanism for these complications is not well defined, maternal insulin resistance has been implicated. Metformin, by reducing insulin resistance, might be expected to improve maternal and foetal outcomes in this condition.

Chiswick and colleagues (2015) reported a double blind, placebo controlled trial (EMPOWaR) in nondiabetic (normal glucose tolerance test) obese (BMI of 30 kg/m^2^ or more) predominantly Caucasian (96%) women receiving metformin 500 mg (up to a maximum of 2500 mg/day) or placebo from 12–16 weeks gestation until term [22].

Three women developed preeclampsia in either group. Fasting plasma glucose and HOMA-IR, measures of maternal insulin resistance, were lower at 28 weeks in women who received metformin but these differences were not maintained at 36 weeks possibly due to poor study drug compliance late in pregnancy. The EMPOWaR investigators also noted significantly lower inflammatory markers including C-reactive protein and Interleukin-6 (IL-6) in women who received metformin. Disappointingly, these changes had no significant effect on the subsequent development of gestational diabetes. The primary outcome, a *z* score corresponding to the gestational age, parity, and sex-standardised birthweight percentile of live-born babies was no different in the offspring of metformin treated women compared to controls [22].

We performed a similar double blind placebo-controlled trial in 450 nondiabetic severely obese (BMI > 35 kg/m^2^) mixed ethnicity (70% Caucasian, 25% African or Afro-Caribbean, 5% South Asian or mixed) women receiving up to 3000 mg metformin daily [23]. Adherence to the study regimen was good (≥50% of tablets taken in nearly 80% of the women) and did not differ significantly between the two groups. Metformin was associated with less maternal gestational weight gain (kg) (median: 4.6 vs. 6.3; *p* < 0.01) and less preeclampsia (6/202 vs. 22/195; *p*: 0.001) compared with the placebo. However, metformin did not reduce the median neonatal birthweight *z* score (primary outcome) or the incidence of gestational diabetes. There was no significant difference between the groups in the incidence of other pregnancy complications or of adverse foetal or neonatal outcomes [23].

There are several possible explanations for the failure of these studies to show an impact on birth weight or gestational diabetes. Firstly, metformin was initiated late in the first trimester and it is possible that a beneficial effect would have been observed if started around the time of conception. Evidence from women with polycystic ovaries who receive metformin pre- or peri-conception, indicates a reduction in the risk of GDM [7]. Secondly, it is possible that a beneficial effect on birthweight in these women requires a high dose of metformin (2500–3000 mg/day) and too few women in these trials took this dose for long enough. Women with obesity in pregnancy may represent a heterogeneous group with varying degrees of resistance to the insulin sensitising effects of metformin. Genetic polymorphisms in drug uptake transporter genes have been implicated as a possible mechanism accounting for variation in metformin response [24].

Another possibility is that the impact of metformin will be seen in early childhood rather than at birth. Beneficial changes in the body composition of children who were exposed to metformin in utero in the MiG study have been reported at 2 years of follow-up (MiG TOFU) [25]. Similar results might be seen in the children of women who participated in EMPOWaR or Metformin in Obese non-diabetic Pregnant women (MOP) trials and such studies are in progress.

**Figure 1:**
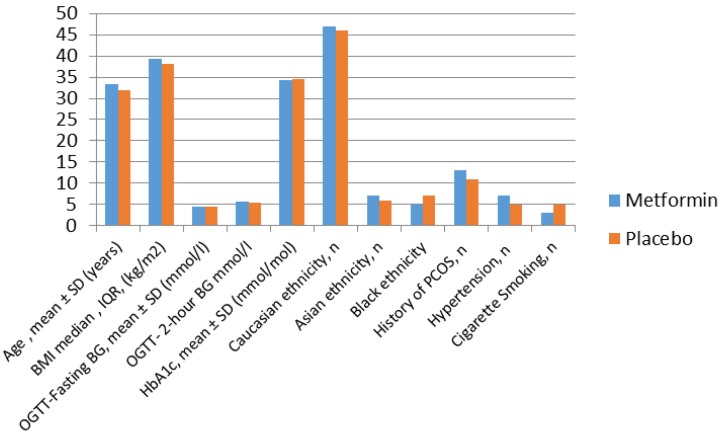
Baseline characteristics of the subset of 118 women randomised to metformin and placebo groups.

We wanted to test the hypothesis that metformin reduces the incidence of GDM and associated features of the metabolic syndrome in women with the highest insulin resistance at baseline. We therefore performed a sub analysis on a subset of 118 patients in whom data on maternal fasting insulin, HOMA-IR, visceral fat mass (VFM) and inflammatory markers was collected. Their baseline characteristics are shown in Figure 1. These 118 women were randomised to either metformin or placebo (59 in each group) [26]. Body composition was assessed at entry to the study, at 28 weeks gestation, at term, and postnatally by the InBody^TM^720 using Direct Segmental Multi-frequency Bioelectrical Impedance Analysis Method (DSM–BIA Method) which has been validated and correlates well with intra-abdominal fat area assessed by CT scan [27] and DEXA [28].

**Figure 2:**
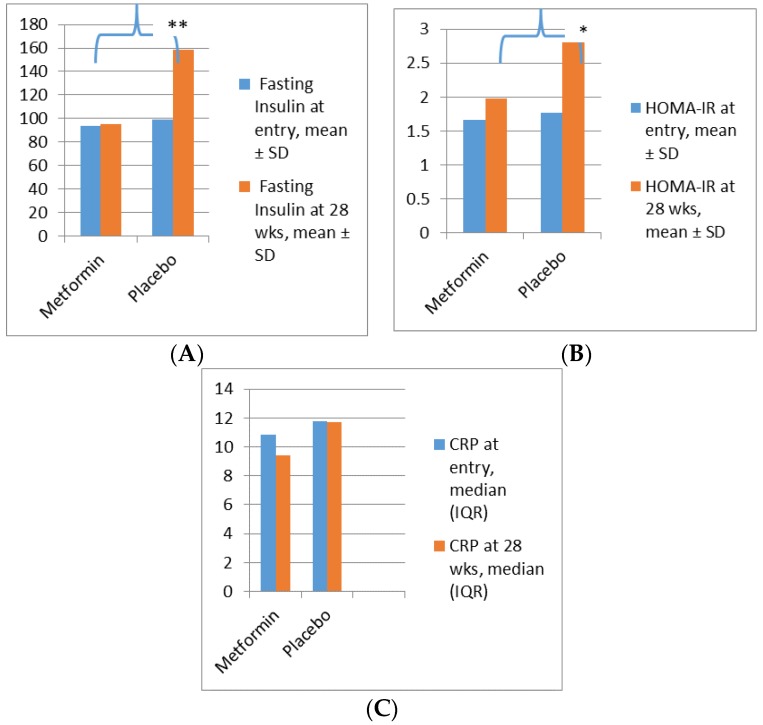
Changes in fasting insulin, HOMA-IR, and C-reactive protein from baseline to 28 weeks gestation. (**A**): Change in fasting insulin between the metformin and placebo groups: ** *p*: 0.009. (**B**): Change in HOMA IR between the metformin and placebo groups: * *p*: 0.03. (**C**): Change in C-reactive protein between the metformin and placebo groups: *p*: NS.

**Figure 3:**
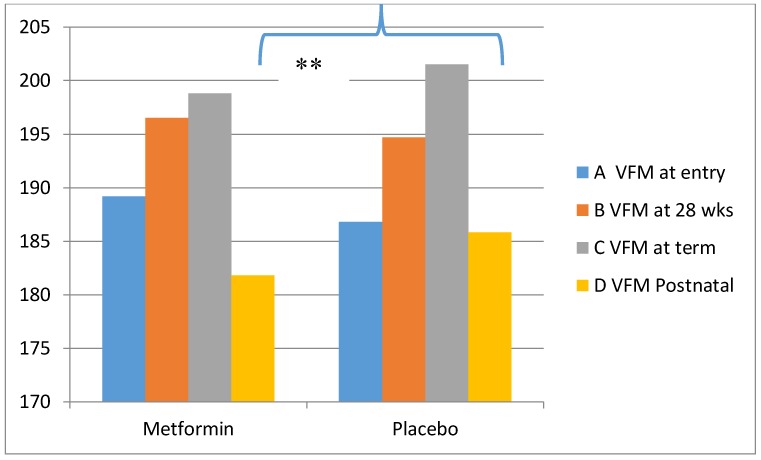
Changes in maternal visceral fat mass at entry (**A**), 28 weeks (**B**), term (**C**) and after pregnancy (**D**) (** p = 0.01).

We found that metformin attenuated the physiological increase in fasting insulin and HOMA-IR at 28 weeks gestation in comparison with placebo (Figure 2). Maternal gestational weight gain (kg) was significantly reduced in the metformin group (3.9 ± 4.6 vs. 7 ± 4.5, *p*: 0.003). Changes in visceral fat mass in the two groups are shown in Figure 3. Whilst the rise in VFM was reduced in women receiving metformin, this did not achieve statistical significance. Postnatally, there was a greater decrease in visceral fat mass in the metformin group (−8.8 ± 15.5 vs. −0.6 ± 15.8; *p*: 0.01).

**Figure 4:**
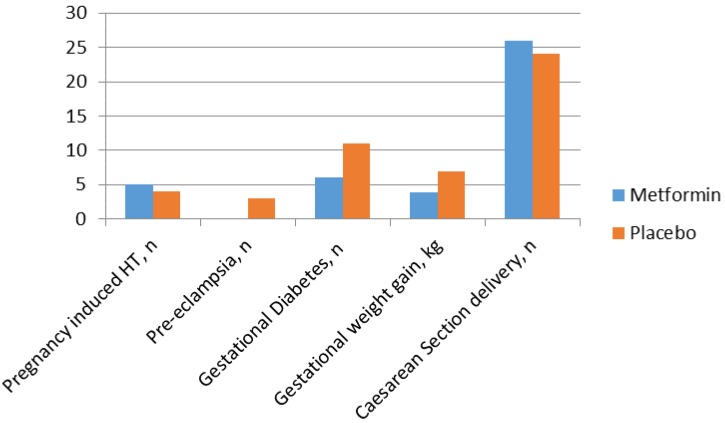
Pregnancy outcomes in women randomised to the metformin and placebo groups.

**Figure 5:**
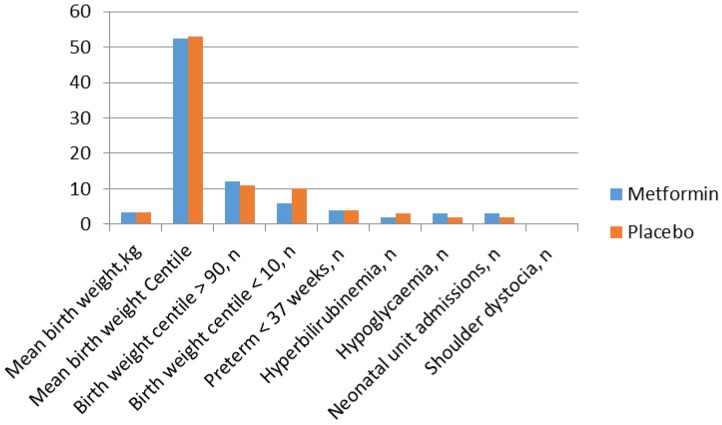
Neonatal outcomes in women randomised to the metformin and placebo groups.

Pregnancy and neonatal outcomes are shown in Figure 4 and Figure 5. When stratified as high (HOMA IR > 75th percentile) or normal insulin resistance, only 1 (6.6%) of the 15 women with high maternal insulin resistance allocated to metformin developed GDM compared with 4 (44.4%) of the nine women with high maternal insulin resistance allocated to placebo (*p*: 0.04). Women with the most severe insulin resistance at entry to the study allocated to placebo had a greatly increased risk of GDM (OR: 5.7 (1.2–27.5)). Nevertheless, the overall incidence of GDM was not significantly reduced by metformin treatment when stratified by baseline maternal insulin resistance. It remains possible that the lifestyle advice offered to both groups diluted a real effect on GDM frequency and that larger numbers may be needed to show a significant difference.

The reduced rate of preeclampsia observed in the MOP trial contrasts with the lack of effect in EMPOWaR. One explanation for this difference is that gestational weight gain was reduced in MOP but not in EMPOWaR [22,23]. Lower maternal weight gain was associated with a reduced rate of preeclampsia in a recent meta-analysis of metformin and risk of preeclampsia [29].

## 4. Adverse Events

There was no significant difference in the incidence of serious adverse events between the groups, but the incidence of side effects like nausea, vomiting, and diarrhoea were higher in the metformin group as compared to the placebo group. Eleven patients receiving metformin and four receiving placebo complained of nausea and vomiting. Similarly, more patients in the metformin group complained of diarrhoea (nine patients) as compared to the placebo group (two patients). In three patients, one in the metformin group and two in the placebo group, foetal scan showed foetal growth restriction with estimated foetal weight < 5th percentile and abnormal foetal Doppler studies. The trial medications were stopped in these patients as per protocol guidelines. The trial medications were started between 12 and 18 weeks of gestation. The percentage of women taking >2500 mg of Metformin per day was an overall 88.1%.

### 4.1. Metformin in Gestational Diabetes

Diabetes in pregnancy may be pre-gestational, which is when a woman with established diabetes becomes pregnant, or gestational, which is traditionally defined as “carbohydrate intolerance of varying severity with onset or first recognition during pregnancy”. The International Association of Diabetes and Pregnancy Study Groups (IADPSG), the ADA and others have recently attempted to distinguish women with probable pre-existing DM that is first recognised during pregnancy (overt diabetes) from transient manifestation of pregnancy related insulin resistance (gestational diabetes) [30]. The prevalence of gestational diabetes (GDM) is increasing worldwide as the pregnant population is becoming older and also as the prevalence of obesity is increasing. Comparisons of prevalence between countries are difficult because different diagnostic criteria are currently adopted [31].

Evidence for the use of metformin in gestational diabetes comes from randomised controlled trials as well as case-control observational studies. The landmark Metformin in Gestational Diabetes (MiG) trial had a major impact on the management of GDM in many countries including in the UK [32]. In this study, women were randomised to either metformin or usual treatment i.e., insulin. A high proportion of women assigned to metformin required supplementary insulin (46%) but at considerably lower doses than women receiving insulin alone. The primary outcome was a composite of neonatal hypoglycaemia (<2.6 mmol/L), respiratory distress, need for phototherapy, 5 min Apgar score < 7 or premature birth (before 37 weeks), and was no different between the two treatment groups [32].

Maternal weight gain from enrolment to terma secondary outcome was significantly less in women taking metformin vs. those on insulin (0.4 ± 2.9 kg in the metformin group vs. 2.0 ± 3.3 kg in the insulin group; *p* < 0.001). Other secondary outcomes including birthweight, neonatal anthropometrics and rates of large for gestational age (>90th percentile) were also similar in the metformin and insulin groups. However, the rates of severe hypoglycaemia (<1.6 mmol/L) were reduced in the metformin group vs. insulin therapy [32].

The MiG trial also found that patient acceptability was much higher for metformin than for insulin; when asked if they would choose it again for subsequent pregnancies, 77% of women on metformin said they would versus only 27% for those on insulin. Gastrointestinal side effects of metformin required 32 women (8.8%) to reduce their dose but only seven (1.9%) had to stop treatment [32].

In the light of the MiG findings, we conducted a case-control observational study comparing pregnancy outcomes in 100 women with GDM exclusively treated with metformin vs. 100 with GDM exclusively treated with insulin and matched for age, weight, and ethnicity [33]. Both groups had similar baseline maternal risk factors. The incidences of gestational hypertension, pre-eclampsia, induction of labour and rate of Caesarean section were similar but, as in the MiG trial, mean maternal weight gain from the enrolment to term was significantly lower in the metformin group. The pregnancy outcomes in the women who were treated with metformin alone, demonstrated lesser incidence of prematurity, neonatal jaundice, and admission to neonatal unit with an overall improvement in neonatal morbidity as compared to the women treated with insulin alone. There was no significant difference in the incidence of foetal macrosomia between the two groups of women [33].

In a further case-control study, we compared outcomes in 324 metformin-treated GDM women with 175 GDM women managed with dietary measures alone and matched for age and ethnicity [34]. Despite greater glucose intolerance and hence increased maternal risk in the metformin group, the proportion of macrosomic babies (birth weight [BW] centile > 90th centile) and small for gestational age (SGA) (BW < 10th centile) in this group was significantly lower than women treated with diet alone (12.7% versus 20%; *p* < 0.05 [macrosomia]; 7.7% versus 14.3% [SGA] *p* < 0.05) [34].

When comparing metformin with other treatments, post-prandial glycaemic levels may be important and it is notable that in a meta-analysis of three randomised controlled studies of GDM patients, lower post-prandial glucose was observed in metformin vs. insulin treated patients although these differences did not achieve statistical significance [35]. In a recent systematic review, metformin did not increase the rate of preterm delivery or Caesarean section, or risk of small for gestational age babies [36]. However, metformin was associated with lower risks of large for gestational age babies, neonatal hypoglycaemia and admission to neonatal intensive care units, as well as reduced rates of pregnancy induced hypertension [36].

In its latest guidance (2015), NICE states “offer metformin to women with gestational diabetes if blood glucose targets are not met using changes in diet and exercise within 1–2 weeks. Offer insulin instead of metformin to women with gestational diabetes if metformin is contraindicated or unacceptable to the woman.” [1].

### 4.2. Metformin for Women with Type 2 Diabetes

Whilst randomised controlled trial evidence assessing the use of metformin in pregnant women with type 2 diabetes is not currently available, clinicians will be very familiar with the clinical scenario of a woman whose diabetes is well controlled on metformin who presents in early pregnancy. In this situation, stopping the metformin risks exposing the foetus to the hazards of hyperglycaemia. Reassuringly no increase in congenital malformations or neonatal mortality have been seen in two meta-analyses of observational studies [37,38]. By contrast, Hellmuth and colleagues (2000) reported increased perinatal mortality and preeclampsia in a retrospective study of 50 women with type 2 diabetes taking metformin compared with those treated with sulphonylureas or insulin [39]. However women taking metformin were more obese than women in the other treatment groups which may have confounded the results. More recently, Hughes and Rowan (2006) reported no difference in maternal and foetal outcomes in women taking metformin compared with those on insulin despite the metformin group being at higher risk at baseline of adverse outcomes [40]. In another observational study, Ekpebegh and colleagues analysed 379 women with type 2 diabetes using oral agents between 1991–2000 in South Africa [41]. The authors found a high perinatal mortality (125 events per 1000 births) in women treated with oral agents (sulphonylureas or sulphonylureas plus metformin) but not with metformin alone. Insulin, by contrast, either after oral agents or after diet, was associated with a low perinatal mortality [41].

Currently, the Canadian multi-centre MiTy trial is assessing the possible benefit of adding metformin to insulin for type 2 diabetes in the first or second trimesters [42]. The primary outcome is a composite neonatal outcome of pregnancy loss, preterm birth, birth injury, moderate/severe respiratory distress, neonatal hypoglycaemia, or neonatal intensive care unit admission longer than 24 h. The trial aims to enrol 500 participants [42].

NICE (2015) recommends that women with pre-existing type 2 diabetes can receive metformin as an adjunct or alternative to insulin in the preconception period and during pregnancy when the likely benefits from improved glucose control outweigh the potential for harm [1]. As the evidence base for treatment expands, this balance will be more clearly defined.

### 4.3. Metformin for Women with PCOS

There is increasing evidence that hyperinsulinaemia and insulin resistance play a central role in the pathogenesis of polycystic ovarian syndrome (PCOS) [43]. Women with PCOS are more insulin resistant than weight-matched women with normal ovaries [44]. Obesity in combination with PCOS reduces the chances of conception and the response to fertility treatment, and increases the risk of miscarriage and GDM [45].

Metformin has an established place in the management of PCOS being used to induce ovulation, reduce miscarriage rates, prevent foetal growth restriction, and improve the metabolic associations such as glucose intolerance [46]. Since metformin may be used pre-conception, it is reassuring that there is no evidence of teratogenicity. A meta-analysis of first trimester exposure to metformin in 351 women found no increase in birth defects [16].

The evidence base for the use of metformin in PCOS is mainly derived from case-controlled studies with occasional conflicting results. An exception is a randomised placebo-controlled trial in which 257 women with PCOS received metformin (500 mg twice daily increasing to 1000 mg twice daily) or placebo from the first trimester to delivery [47]. The investigators found no difference in the primary outcome, a composite of preeclampsia, GDM and preterm delivery [47].

Metformin may need to be started pre-pregnancy and be given at higher doses to reduce pregnancy related complications. Thus, De Leo and colleagues (2011) in a prospective study of 98 women with PCOS in whom metformin (1700–3000 mg/day) was started before conception and continued until 37 weeks of pregnancy, reported a significant reduction of pregnancy complications, such as gestational diabetes and gestational hypertension compared with 110 pregnant controls [48]. The decrease in preeclampsia was not statistically significant and mean neonatal Apgar scores, weight and length were similar. between the two groups [48]. Reductions in pregnancy related complications were also demonstrated by Khattab et al. (2011) who compared 200 nondiabetic PCOS women who had conceived on metformin and continued it (1000–2000 mg/day) throughout pregnancy with 160 women who has similarly conceived on metformin but discontinued it when finding themselves pregnant [49]. The investigators found a statistically significant reduction in preeclampsia (OR: 0.35, 95% CI: 0.13–0.94) and GDM (OR: 0.17, 95% CI: 0.07–0.37) in those who continued metformin [49]. In another study, continuation of metformin throughout pregnancy resulted in reduced rates of foetal growth restriction, GDM, pre-term labour and increased live births [50]. No congenital anomalies, intrauterine deaths, or stillbirths were reported.

Metformin may also reduce early pregnancy losses. A meta-analysis of randomised controlled trials comparing ovulation induction treatments concluded that clomiphene plus metformin was more effective than clomiphene alone in terms of ovulation and pregnancy [51].

### 4.4. Potential Impact for Children after in Utero Exposure to Metformin

Could the use of metformin in pregnancy exert long term beneficial, neutral, or deleterious effects on the offspring? At present, a definitive answer cannot be given. Evidence suggests that intrauterine exposure to the hyperglycaemia of diabetes poses an increased risk of childhood obesity and diabetes in later life, over and above any risk attributable to genetic factors [52,53]. These infants are insulin resistant [54]. It could therefore be postulated that insulin resistance resulting from epigenetic changes and foetal programming could be reversed by metformin.

A hint that this might indeed be the case comes from follow-up of infants of women in the MiG study examined at 2 years of age [25]. The metformin exposed infants had increased subcutaneous fat as assessed by subscapular and biceps skinfolds in comparison with non-exposed infants, whilst total body fat was similar. The MiG investigators hypothesized that this represents a healthier fat distribution [25]. If indeed, these children were shown to have less visceral fat, they would be expected to be more insulin sensitive.

Longer term studies are clearly needed. Eight year follow-up of 12 children of PCOS mothers exposed to metformin during pregnancy showed increased fasting glucose, systolic blood pressure, and lower Low Density Lipoprotein (LDL) compared with placebo [55]. The significance of these findings is unclear given the small numbers.

Recently the MiG trialists reported body composition and metabolic outcomes at 7 years (109/181 children in Adelaide) and 9 years (99/396 children in Auckland) of age [56]. At 7 years, there were no differences in offspring measures.

At 9 years, metformin exposed children had increased weight, arm and waist circumferences, waist: height (*p* < 0.05), body mass index, triceps skin fold (*p*: 0.05), DEXA fat mass and lean mass (*p*: 0.07) [56]. Body fat percentage was similar by DEXA and bioimpedence. Visceral adipose tissue and liver fat were similar by Magnetic Resonance Imaging (MRI). Metabolic markers including HbA1c, fasting glucose, fasting lipids, adiponectin, and leptin were all similar. The significance of these findings in terms of long term cardiovascular risk is uncertain.

It is important to note that the effects of diabetes in pregnancy on childhood obesity may not become manifest until after aged 6–9 years [57]. Planned follow-up of the offspring in the EMPOWaR and MOP trials may shed more light as these studies compared metformin with placebo rather than insulin. The MiTy Kids trial will assess the children of mothers with type 2 diabetes receiving metformin in addition to insulin.

## 5. Summary

Metformin in pregnancy does not increase congenital abnormalities and is generally well tolerated. Serious side-effects are very rare. In GDM, because it reduces maternal weight gain compared with insulin, metformin is now the preferred option if glucose targets are not met with dietary measures. This is especially the case for the increasing number of women who are obese. It can be safely added to insulin for women inadequately controlled on insulin alone and allows lower doses of insulin to be used.

For women with type 2 diabetes in pregnancy, the risks are significantly higher than in GDM. The evidence base for decision making is more limited but in the absence of evidence favouring insulin, metformin should be continued for women already established on it. Insulin may need to be added if glucose targets are not achieved. We await with interest the results of the ongoing MiTy trial.

Metformin started before pregnancy and continued until term in women with PCOS has benefits both for the mother (reducing GDM, gestational hypertension, preterm labour) and the developing foetus (reducing early pregnancy loss, foetal growth retardation).

At present, we do not recommend metformin for nondiabetic pregnant women with obesity. We do not know whether preconception use of metformin in larger doses would have been effective in reducing birth weight centiles. The reduction in preeclampsia seen in the MOP trial is intriguing and needs further study.

The largely unanswered question is the long term impact of intrauterine metformin exposure on childhood development. The MiG TOFU results at 9 years could be interpreted as showing a neutral effect as body fat, visceral adipose tissue, and liver fat were similar in metformin and insulin groups. Conversely the unexpected finding of increased body mass index in the metformin offspring might indicate an increased risk of childhood obesity. The low follow-up rate, however, makes the results difficult to interpret. On-going long term follow-up studies including from the offspring of mothers in the obesity trials will help answer this current uncertainty.
